# The sleep EEG spectrum is a sexually dimorphic marker of general intelligence

**DOI:** 10.1038/s41598-017-18124-0

**Published:** 2017-12-22

**Authors:** Péter P. Ujma, Boris N. Konrad, Ferenc Gombos, Péter Simor, Adrián Pótári, Lisa Genzel, Marcel Pawlowski, Axel Steiger, Róbert Bódizs, Martin Dresler

**Affiliations:** 1 Institute of Behavioural Sciences, Semmelweis University, H-1089 Budapest, Hungary; 2 Donders Institute for Brain, Cognition and Behaviour, Radboud University Medical Centre, 6525 EN Nijmegen, The Netherlands; 3 National Institute of Clinical Neuroscience, H-1145 Budapest, Hungary; 4 Nyírő Gyula Hospital, National Institute of Psychiatry and Addictions, H-1135 Budapest, Hungary; 5 Department of Cognitive Sciences, Budapest University of Technology and Economics, H-1111 Budapest, Hungary; 6 Centre for Cognitive and Neural Systems, University of Edinburgh, EH8 9JZ Edinburg, United Kingdom; 7 Max Planck Institute of Psychiatry, 80804 Munich, Germany

## Abstract

The shape of the EEG spectrum in sleep relies on genetic and anatomical factors and forms an individual “EEG fingerprint”. Spectral components of EEG were shown to be connected to mental ability both in sleep and wakefulness. EEG sleep spindle correlates of intelligence, however, exhibit a sexual dimorphism, with a more pronounced association to intelligence in females than males. In a sample of 151 healthy individuals, we investigated how intelligence is related to spectral components of full-night sleep EEG, while controlling for the effects of age. A positive linear association between intelligence and REM anterior beta power was found in females but not males. Transient, spindle-like “REM beta tufts” are described in the EEG of healthy subjects, which may reflect the functioning of a recently described cingular-prefrontal emotion and motor regulation network. REM sleep frontal high delta power was a negative correlate of intelligence. NREM alpha and sigma spectral power correlations with intelligence did not unequivocally remain significant after multiple comparisons correction, but exhibited a similar sexual dimorphism. These results suggest that the neural oscillatory correlates of intelligence in sleep are sexually dimorphic, and they are not restricted to either sleep spindles or NREM sleep.

## Introduction

Intelligence, as expressed by the results of standardized IQ tests such as Raven Advanced Progressive Matrices (RAPM) or the Culture Fair Test (CFT), is a statistically and temporally stable trait with a great degree of heritability^1–3^. Such characteristics would imply a strong, well-defined biological basis, however, the precise mechanisms through which IQ is implemented in the nervous system remain elusive^4–6^, and the search for potential biological markers is still ongoing.

In recent years, an increasing body of research suggests EEG sleep spindles as a biological marker of intelligence^7–14^, however the methodology of these studies is quite heterogeneous and their results not always consistent^15^. Our previous research^15–17^ confirmed that sleep spindles – particularly sleep spindle amplitude – are indeed positively correlated with IQ, although in a sexually dimorphic manner: in children, adolescents as well as adults, sleep spindle parameters are preferentially associated with IQ in females.

Some evidence suggests that sleep spindles are not the only potentially relevant electrophysiological biomarkers of IQ. First, a relationship between intelligence and wake EEG features such as alpha power, event-related alpha desynchronization and coherence has been described^18–22^. Second, one study^7^ investigated the correlates of IQ not with individually detected sleep spindle events, but with NREM sleep EEG spectral power, and indeed found an association between IQ and spectral power well outside the conventional sleep spindle frequency range, specifically with theta, alpha and beta power. Uniquely, this study also analyzed REM sleep EEG power, and found similar associations with IQ.

Sleep spindles were conceptualized as potential correlates of IQ because of the existence of the sleep EEG spectral fingerprint^14,23,24^, that is, the inter-individual variability, intra-individual stability and genetic determination of the shape of the NREM sleep EEG spectrogram. The reliability of the sleep EEG spectrogram, approximated by within-subject internight correlations, is very high, typically around 0.9 (Tan *et al*., 2000; Tan *et al*., 2001; Van Dongen *et al*., 2005). It must be pointed out, however, that these reliability estimates were usually obtained using the sleep EEG recordings of healthy, young subjects: therefore, a potential effect of age, sex, minor illnesses or other confounders on the reliability of the sleep EEG spectrogram is not known. Originally, the high reliability of the sleep EEG spectrogram was demonstrated well outside the sigma range in NREM^25–29^. Notably, the descriptive parameters^30,31^ as well as EEG characteristics^32,33^ of REM sleep are similarly stable and heritable. Therefore, the scarcity of data on the relationship between IQ and sleep EEG features other than sleep spindles is somewhat surprising.

We hypothesized that REM and NREM spectral features of the sleep encephalogram also outside the sigma range are good candidate markers of IQ due to their individual stability and genetic determination. To test this hypothesis, we computed EEG spectral power from a large sample of subjects (N = 151) and investigated the association of a broad range of REM and NREM EEG spectral features with measurements of intelligence in order to find other potential IQ markers in the sleep EEG fingerprint. Given the sexually dimorphic association between sleep spindles and IQ^15–17^, we expected a similar sexual dimorphism also for the relationship between EEG spectral power and IQ.

## Materials and Methods

Polysomnography data from 151 subjects (68 females, 83 males, mean age 29.3 years, age range 17–69 years) was analyzed in this study. Another 9 subjects were excluded due to heavily artifact-contaminated data. Data was combined from multiple databases (Max Planck Institute of Psychiatry, Munich, Germany; Institute of Behavioural Sciences of Semmelweis University, Budapest, Hungary) for this retrospective multicenter study^15,34^. The research protocols were approved by the Ethical Committee of the Semmelweis University, Budapest, or the Ludwig Maximilian University, Munich; and research was carried out in accordance with the Declaration of Helsinki. All subjects signed informed consent for the participation in the studies. According to a semi-structured interview with experienced psychiatrists or psychologists, all subjects were healthy, had no history of neurologic or psychiatric disease, and were free of any current drug effects excluding contraceptives. Consumption of small habitual doses of caffeine (max. 2 cups of coffee before noon), but no alcohol was allowed before the recordings. 6 male and 2 female subjects were light to moderate smokers (self-reported), while the rest of the subjects were non-smokers.

Sleep was recorded for two consecutive nights by standard polysomnography, including EEG according to the 10–20 system (recordings sites: Fp1, Fp2, F3, F4, Fz, F7, F8, C3, C4, Cz, P3, P4, T3, T4, T5, T6, O1, and O2, re-referenced to mathematically linked mastoids), electro-oculography (EOG), bipolar submental electromyography (EMG), and electrocardiography (ECG). Impedances for the EEG electrodes were kept below 8 kΩ. Supplementary Table 1 shows further recording details including the precise distribution of subjects by study center. Due to electrode failure, data from a total of 26 electrodes from 21 subjects was excluded and was treated as missing data in all subsequent analyses. Electrode failures occurred on Fp1 in 10 cases; Fp2 in 3 cases; F4, F8, F7 and T5 in 2 cases; F3, T3, C3, O2 and T6 in 1 case, respectively.

Visual stage scoring on a 20 second basis by according to standard criteria^35^ and visual artifact rejection on a 4 second basis was performed on sleep EEG recordings from the second laboratory nights. Artifact-free NREM2 and SWS epochs were analyzed to obtain NREM spectral data, and artifact-free REM epochs were analyzed to obtain REM spectral data. Spectral analysis was performed by the mixed-radix FFT method using 4 s Hanning-tapered windows with a 2 s overlap and averaging power spectral densities from all 4 s windows. Power spectral densities were calculated for 0.25 Hz bins from 0 Hz to the Nyquist frequency (sampling rate/2). In all analyses, relative log-transformed power spectral density from 1 Hz to 40 Hz was used. This was calculated by dividing the amplitude reduction corrected power of each frequency bin of each electrode of the corrected spectra in the 1–40 Hz range by the sum of power in all frequency bins in this range, and then replacing each value by its 10-base logarithm.

EEG recording devices have different analog filter characteristics which results in different machines yielding different spectral power densities for the same recording, but it is possible to control for such discrepancies^36^. We connected an analog waveform generator to the C3 and C4 electrode inputs (with original recording reference, re-referenced for A1-A2 common references for further analysis) of all EEG devices and applied 40 and 355 μV amplitude sinusoid signals of various amplitudes (0.05 Hz, every 0.1 Hz between 0.1–2 Hz, every 1 Hz between 2–20 Hz, every 10 Hz between 10 Hz-100 Hz). The amplitude reduction rate of each recording system at each frequency was determined by calculating the proportion between digital (measured) and analog (generated) amplitudes of sinusoid signals at the corresponding frequency. Amplitude reduction rates calculated from 40 and 355 μV signals were averaged for each frequency, and amplitude reduction rates for intermediate frequencies were calculated by spline interpolation using frequency responses at neighboring frequencies. Power spectral density values of all frequencies were corrected by dividing the original value by the squared amplitude reduction rate of the appropriate recording device at the corresponding frequency.

All subjects completed one or two standardized nonverbal intelligence tests, the Culture Fair Test (CFT) or Raven Advanced Progressive Matrices (Raven APM), similar tests of abstract pattern completion which yield strongly correlating results and which are particularly good measures of the general factor of intelligence^37–39^. A total of 110 subjects completed the CFT and 81 subjects completed the Raven APM test. 41 subjects completed both tests. A composite raw intelligence test score was calculated, expressed as a Raven equivalent score (RES). RES for Raven APM tests were equal to the actual raw test score. RES of CFT raw scores were equal to the Raven APM score corresponding to the IQ percentile derived from CFT performance and the age of the subject. In case of Raven APM and CFT scores both being available, RES were averaged. The 1993 Des Moines (Iowa) standardization of APM was used.

Pearson’s partial correlation coefficients (correcting for age) were calculated to determine the relationship between power spectral density in each frequency bin of each electrode and RES. Since data from neighboring electrodes and frequency bins are expected to be strongly correlated, rendering a Bonferroni correction overly conservative, multiple comparison correction was performed using the Rüger area method^17,40–42^. In this method, areas of significance are determined along both the spatial and the frequency domain. A Rüger area of potential significance extends from the first frequency bin in which a statistical test is significant on any electrode to the last frequency bin in which a statistical test is significant on any electrode. Areas of potential significance in which the effect was present only on a single electrode or in less than 4 frequency bins (<1 Hz) were not considered in order to eliminate the confounding effects of narrow-frequency artifacts or outlier values. If p < 0.05/2 (p < 0.025) for at least 50% of significant results, or if p < 0.05/3 (p < 0.016) for at least 33% of significant results within this area, then the area is considered statistically significant.

## Results

**Figure 1:**
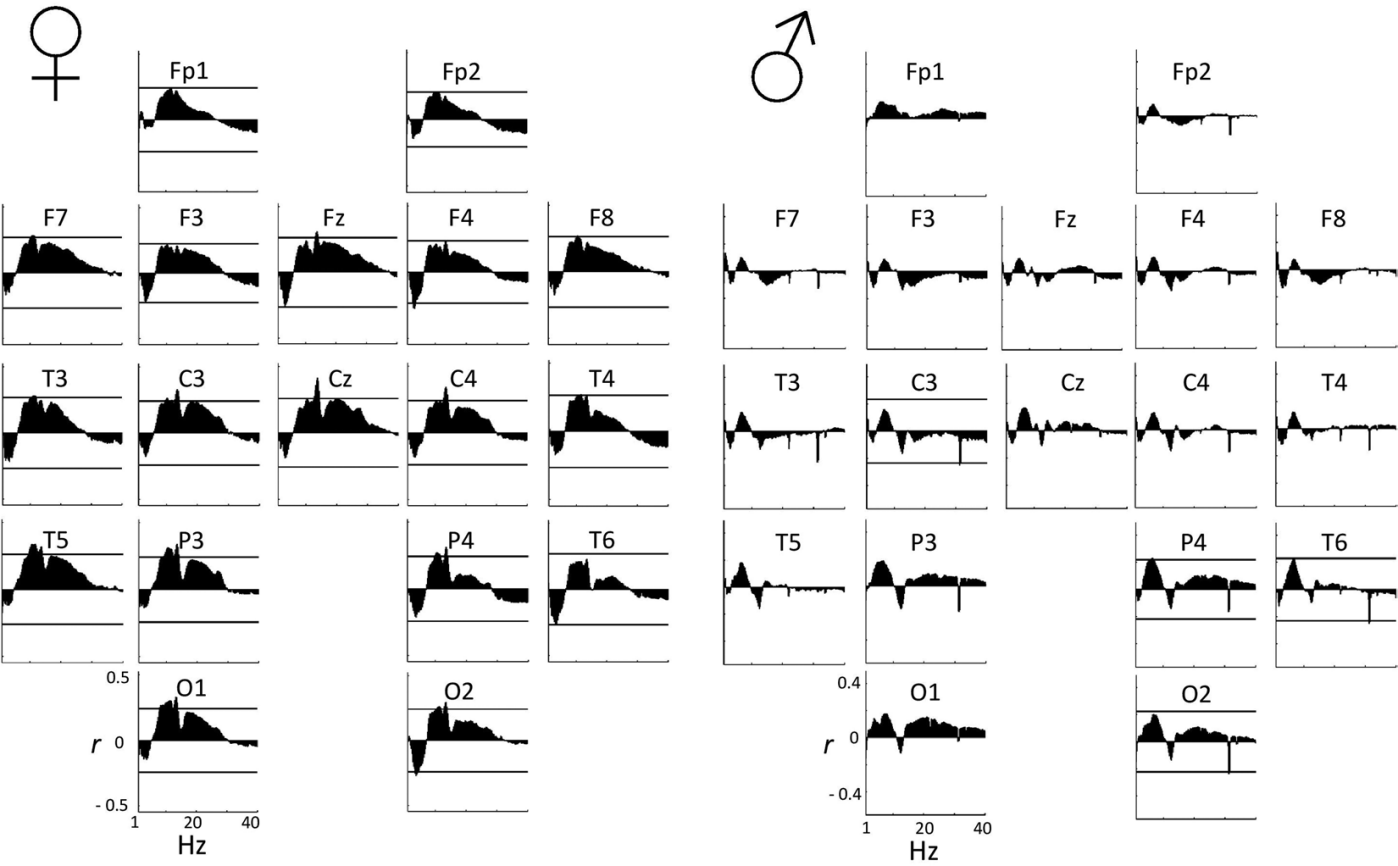
Spectro-correlograms of the age-corrected relationship between relative REM EEG power spectral density (by 0.25 Hz bins) and RES by electrode. Axis X represents frequency between 1 Hz and 40 Hz, while axis Y shows the partial Pearson correlation coefficient between RES and relative EEG power in the given bin, corrected for the effects of age. A horizontal line indicates the critical partial correlation coefficient (p = 0.05) if at least one significant correlation is present on the given electrode.

In REM sleep in females, a significant negative partial correlation with delta/theta power and a significant positive partial correlation with beta power was apparent. Both delta/theta (2.25–5.25 Hz, p < 0.05/2 79.3%, p < 0.05/ 67.4%, maximum association at 3.5 Hz on F7) and beta (10.25–26.75 Hz, p < 0.05/2 63%, p < 0.05/3 38.3%, maximum association at 18.75 Hz on Fz) formed significant Rüger areas (see Fig. 1).

**Figure 2:**
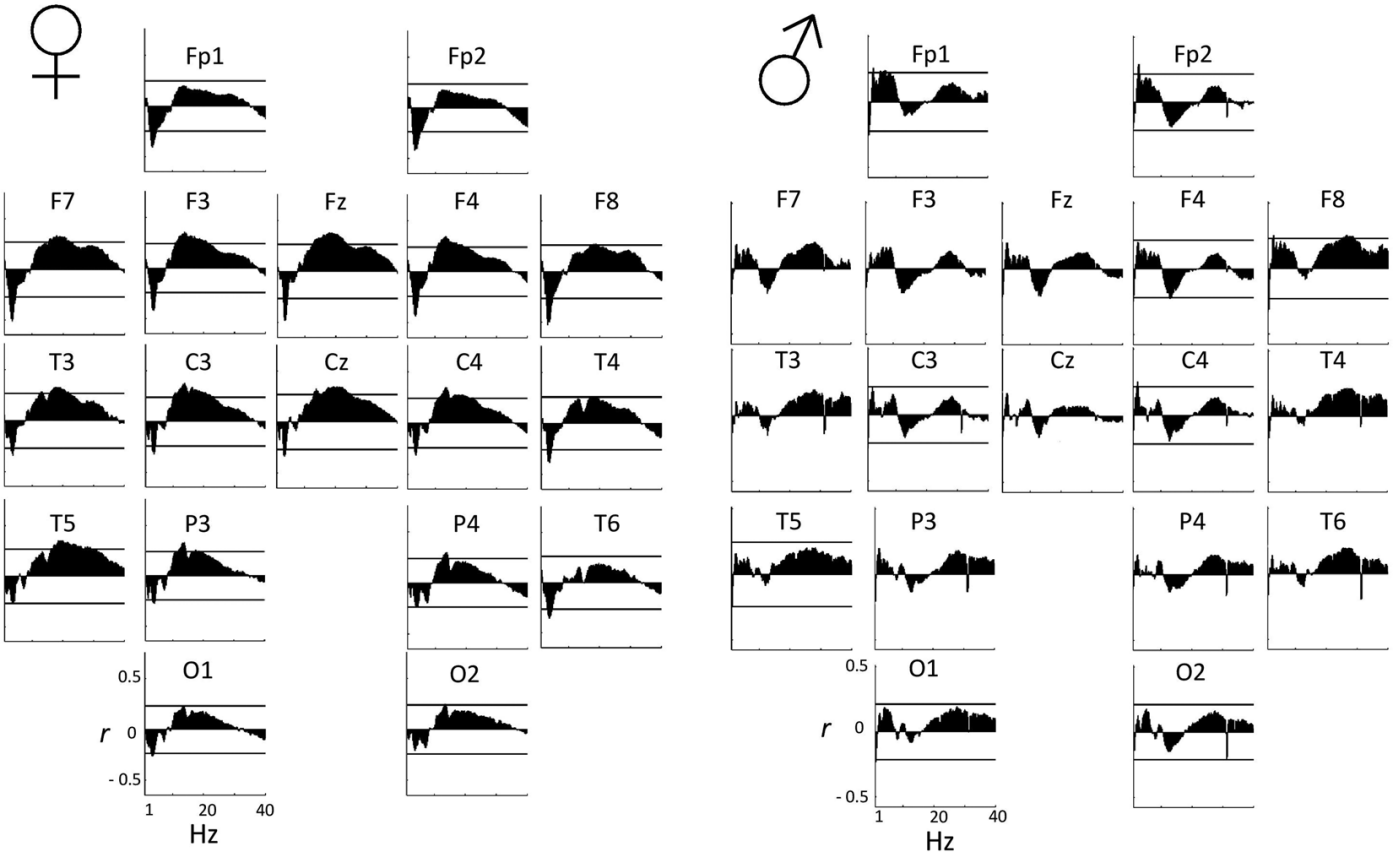
Spectro-correlograms of the age-corrected relationship between relative NREM EEG power spectral density (by 0.25 Hz bins) and RES by electrode. Axis X represents frequency between 1 Hz and 40 Hz, while axis Y shows the partial Pearson correlation coefficient between RES and relative EEG power in the given bin, corrected for the effects of age. A horizontal line indicates the critical partial correlation coefficient (p = 0.05) if at least one significant correlation is present on the given electrode.

In NREM sleep in females, age-corrected partial Pearson correlation coefficients between RES and NREM alpha and sigma power were positive and significant. The associations consisted of two effects overlapping in both the spatial and the frequency domain (Fig. 2) with a posterior, left-lateralized alpha (maximum independent association 11.75 Hz on T5) and a midline sigma effect (maximum association at 13.75 Hz on Cz). However, the overall Rüger area fell slightly short of significance (8–15 Hz, p < 0.05/2 44.44%, p < 0.05/31.11%).

**Figure 3:**
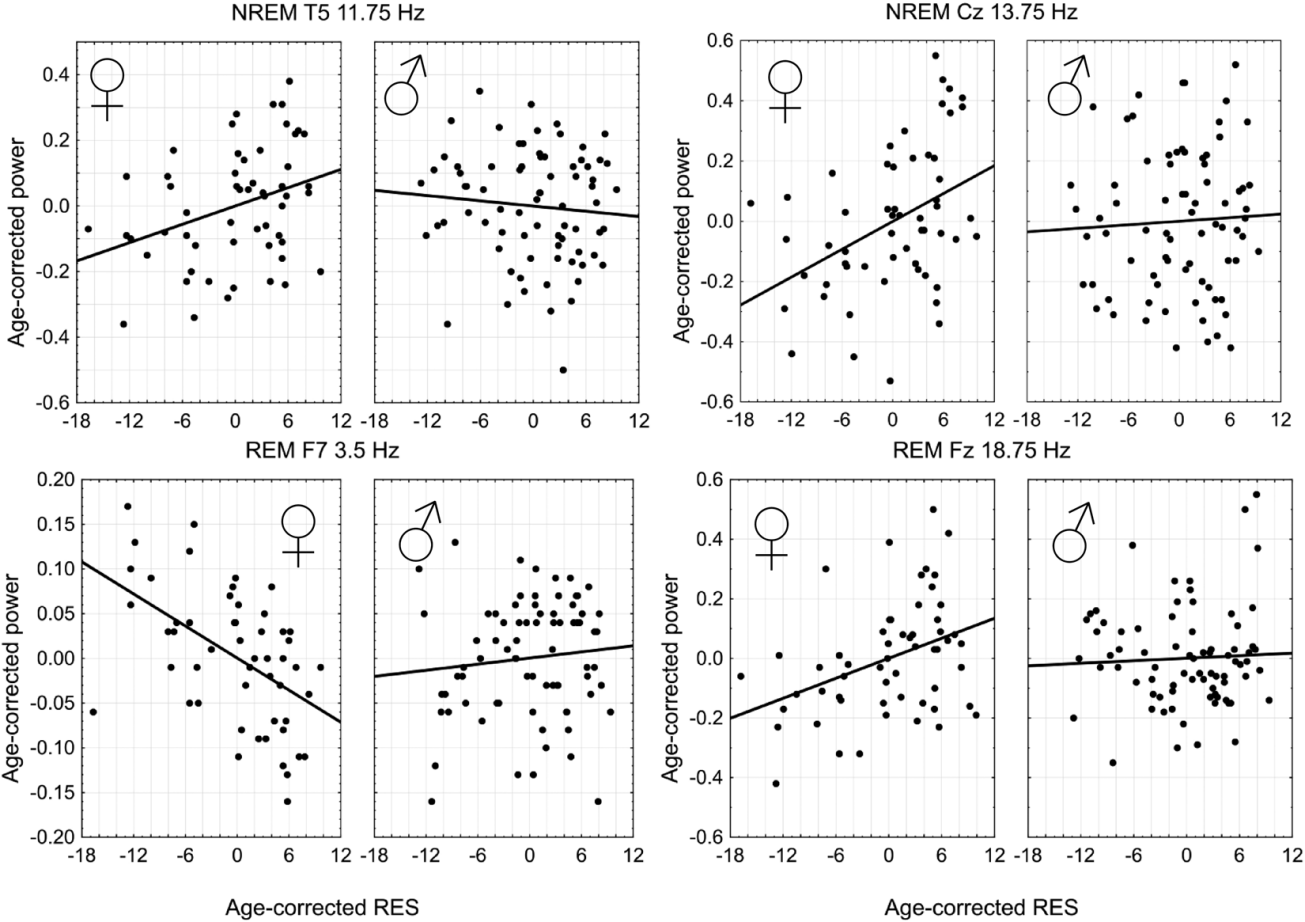
Scatterplots illustrating the age-corrected relationship between RES and relative EEG power for each of the four maximal associations within Rüger areas of potential significance, at the frequency and derivation where the absolute value of the partial correlation coefficient was maximal, separated by sex. Data points indicate the unstandardized residuals of RES (axis X) and relative power (axis Y) after regressing for the effects of age, as the Pearson correlation of these values is equal to the age-corrected partial correlation coefficient of the original values.

**Figure 4:**
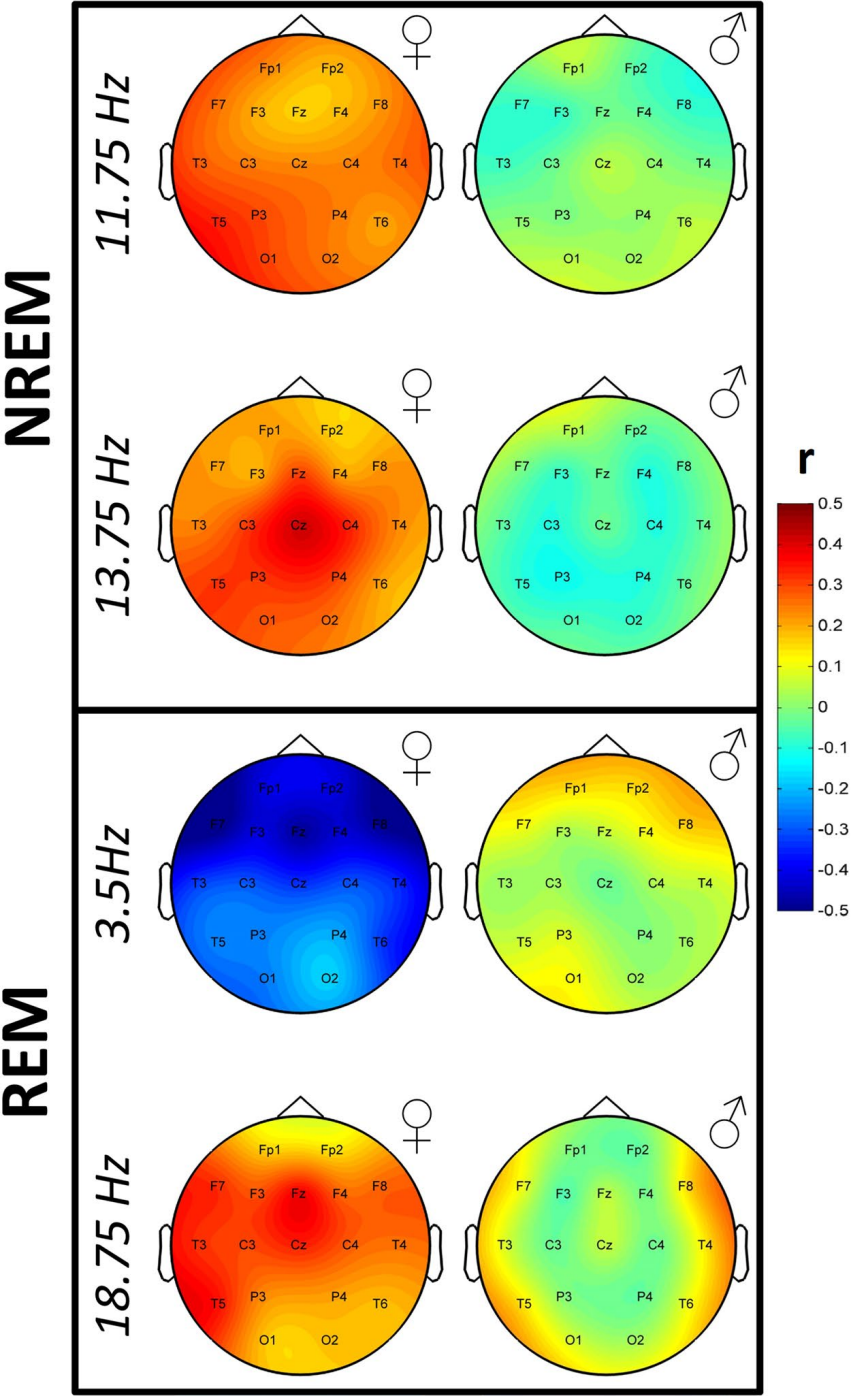
The topographical distribution of association strength at the frequencies of maximal association illustrated on Fig. 3 in females (left column) and males (right column).

In males, no Rüger significant correlations emerged either in REM or NREM sleep. Figure 3 illustrates the maximal partial correlations between relative spectral power and RES in both sexes, while Fig. 4 illustrates the topographical distribution of these associations.

In order to provide evidence for different effect sizes in male and female subjects, we compared the correlation coefficients of each area of potential significance at the derivation and frequency where the association was maximal using Fisher’s r-to-z method. We also considered associations within the NREM Rüger area which fell short of significance because of the strong absolute effect sizes and an interest in the discovery of sexually dimorphic effects. These comparisons were significant in three cases (NREM 13.75 Hz on Cz: z = 2.22, p = 0.0264; NREM 11.75 Hz on T5: z = 2.43, p = 0.0151; REM 3.5 Hz on F7: z = −3.67, p = 0.0001), but remained a trend in the fourth (REM 18.75 Hz on Fz: z = 1.92, p = 0.0549).

In order to test the robustness of our corrected results, we implemented an alternative, permutation-based method of multiple comparisons correction^43^, using the script made available at https://www.mathworks.com/matlabcentral/fileexchange/34920-mult-comp-perm-corr. We obtained age-corrected values of both RES and spectral power by linearly regressing these values against age and calculating the residuals, and performed 5000 permutations across subjects and electrodes for each frequency bin in order to obtain the p-values corrected for family-wise error. As in our original analysis, we performed this correction separately for NREM and REM sleep data, as well as for males and females.

Since this method is sensitive to missing data, we only used spectral data from 12 channels: the 10 electrodes common across all subjects (F3, F4, C3, C4, P3, P4, O1 and O2) and Fz and Cz, which were imputed for subjects without these electrodes as the average spectral value of F3-F4 and C3-C4, respectively. Any other missing channels in other subjects were imputed using the average spectral values at neighboring channels in a similar manner. Electrodes in the temporal arc (F7, F8, T3, T4, T3 and T6) were not used due to the difficulty of interpolating data for these electrodes in the absence of neighboring channels in all but one direction.

This alternative method yielded similar results to the original analysis. In females the correlation between IQ scores and NREM spectral power were significant between 10.25–12 Hz (P3, O1) and 13.25–14 Hz (C3, C4, Cz, P3, O1, O2), while the correlation between IQ scores and REM spectral power was significant between 3.25–4.75 Hz (Fp1, Fp2, F3, F4, Fz, C3, C4) and 12.25–19.5 Hz (F3, F4, Fz, C3, C4, Cz, P3, P4).). (Significant frequency ranges are reported here as the contiguous series of frequency bins in which the corrected association is significant for at least one channel. Channels are mentioned as long as they have at least one significant corrected association within a given frequency range. As in the Rüger area method, we ignored significant frequency ranges with a significant effect of only one electrode. There was one such frequency range in the REM sleep of males where a negative association with 2.75 and 3 Hz spectral power on Fp1 was significant).

This method, unlike the Rüger area approach, reported the female NREM alpha-sigma association as significant, and all our maximal correlations represented on Fig. 3 (11.75 Hz and 13.75 Hz for NREM, 3.5 Hz and 18.75 Hz for REM, all in females only) fell within the range of significant corrected associations with this new method. Supplementary Figure S1 reports adjusted p-values in detail.

## Discussion

Our results demonstrate that spectral characteristics of sleep EEG are indeed related to measures of intelligence, and these associations are not limited to the sigma range: We found significant associations between RES and both low (2.25–5.25 Hz) and high (10.25–26.75 Hz) frequency activity in REM sleep, the former of which exhibited statistically significant sexual dimorphism. NREM alpha and sigma associations with RES were significantly stronger in females than males, but only reached statistical significance using one of the two methods of multiple comparisons correction. Due to the generally high intra-individual reliability of sleep EEG spectral features^29^, it is unlikely that these effects are the result of sex differences in sleep EEG spectrum reliability – that is, lower reliability in males, resulting in the failure of detecting the same association present in females – but in the absence of both previous studies concerning this question and EEG data from multiple nights in our own dataset this remains a theoretical possibility.

### REM sleep

**Figure 5:**
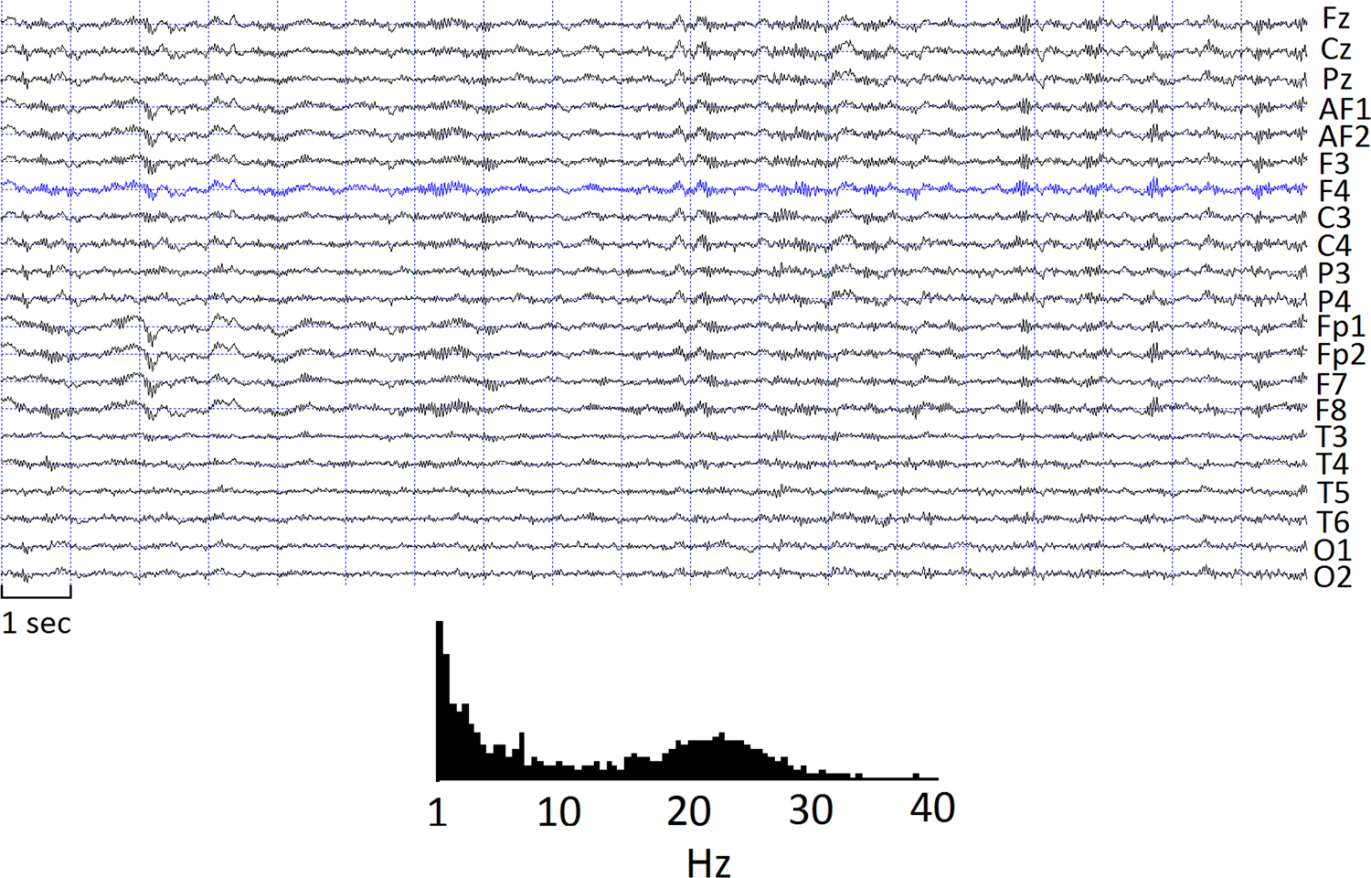
Top: Sample EEG in REM sleep, showing transient bouts of beta activity (“REM beta tufts”). Bottom: spectral composition of the highlighted signal (frequency in Hz, spectral power in arbitrary units).

We wish to use REM beta activity – according to our results, a positive correlate of general intelligence – as an example of a functionally important, but severely under-researched oscillation. Beta EEG activity is an inherent part of the electrical activity patterns of cortical structures in REM sleep^44–46^. The relationship between REM beta activity and cognitive performance has been previously shown in patient populations^47–49^. However, beta activity in REM sleep often does not appear as background activity (comparable to wakefulness), but rather in transient bouts not unlike sleep spindles (see Fig. 5). Such “REM beta tufts” are often readily visible in the EEG and they are obvious contributors to the REM beta spectral power which was shown here to correlate with general intelligence.

Similar beta oscillations have recently been described to be visible over the anterior cingulate and the dorsolateral prefrontal cortex in a small clinical sample with cortically implanted electrodes^50^. The authors speculate that these cortical areas are active during REM sleep and contribute to cognitive functions such as motor memory consolidation and emotion regulation. While our data are not suited to directly test this hypothesis, the reported frontal topography, frequency range and visual appearance of these oscillations is very similar to what we found in the scalp EEG recordings of healthy subjects, and their association with general intelligence highlights their functional importance for cognition.

### NREM sleep

Only one previous study exists about the NREM spectral power correlates of IQ outside the sigma range^7^. While the authors do not attempt to explain this relationship in detail and express doubts about the generalizability of this finding to adult populations, we found a similar relationship in our sample spanning a wide age range, even though it was significant using only one of the two methods of correcting for multiple comparisons. Notably, the tendencies observed in our study were significantly larger in females compared to males and exhibited a maximum at approximately 13.5 Hz, in line with our previous results about the sexually dimorphic relationship between sleep spindles and IQ^15–17^. However, this association was only significant according to the bootstrapping method for multiple comparisons correction, and not using the Rüger areas. The Rüger area is imperfectly suited to disentangle separate effects which smoothly morph together along both the frequency and the spatial domain as it was the case here, evidenced by the presence of two separate areas (alpha and sigma) of association using the bootstrapping method. NREM associations must be interpreted very carefully due to the lack of agreement on their significance across correction methods.

### Sleep quality

Tentatively, one may assume that the significant association between IQ and REM beta power and the tendency-level association with NREM alpha power are both indicative of the same phenomenon: a decrease in sleep quality. High frequency activity in both NREM and REM sleep is generally interpreted as a sign of poor sleep quality^51^ or a sensitive marker of sleep fragility and environmental awareness during sleep^52^, and it was found in patients with insomnia^53,54^ or fibromyalgia^55,56^. However, the function and significance of NREM alpha activity may depend on its topography^51^, and REM beta has also been associated with positive outcomes, that is, lower nightmare severity and more benign PTSD symptoms^57^. Furthermore, low-frequency and beta activity in REM sleep are inversely associated^44–46^, and a pattern of less low frequency and more beta activity, like we observed in the present study, is associated with better functioning in Alzheimer’s disease, potentially reflecting more intact cholinergic pathways^47,49,58^. It is notable that sleep macrostructure parameters were not significantly correlated with RES in either sex and with or without correcting for the effects of age. In sum, there is little evidence that higher NREM alpha and REM beta and lower REM delta in more intelligent female subjects is a sign of poor quality, but it can be speculated that it is associated with an information processing network which is also affected in neurodegenerative diseases. Unfortunately, the lack of previous studies describing the functional importance of the non-sigma elements of the sleep EEG fingerprint associated with intelligence hinders a more detailed interpretation of our findings.

### General perspective

Despite its excellent psychometric properties^59–61^ and importance for a great variety of life outcomes not limited to the cognitive domain^62–64^ the physiological underpinnings of general intelligence remain elusive. Some of the generally replicated physiological parameters associated with intelligence are head/brain size^65^, neural efficiency^20^, sleep spindle measures^15^ and structural and functional imaging properties of widespread cerebral areas^5^, including connectivity patterns^66^, in approximately descending order of the reliability of association. The elusive nature of general intelligence is perhaps best highlighted by genetic studies, which are important not only because they explain the difficulty of finding a single physiological mechanism behind intelligence, but also because they tend to have the greatest statistical power with sample sizes typically in the tens or hundreds of thousands.

General intelligence is very strongly heritable^3,67^, with twin study heritability estimates for adult intelligence of 0.6–0.8. This high heritability indicates that genetically regulated biological functions are a good target for finding putative mechanisms behind general intelligence. However, genome-wise association studies (GWAS) only revealed statistically significant SNPs associated with intelligence^68,69^ or the strongly correlated years in education^70,71^ when extremely large sample sizes were used, indicating that even the strongest single genetic variants have very small effects. Genome-wise complex trait analysis (GCTA)^72–75^ confirms the strong heritability of intelligence, but also the fact that this heritability is the largely additive total effect of many genetic variants with extremely small individual effects, including family-specific rare genetic variations with negative effects^76–78^. Furthermore, the genetic variants associated with intelligence are highly pleiotropic, evidenced by the strong genetic correlation between intelligence and phenotypically correlated traits, such as LDL cholesterol levels, the risk of diabetes and cardiovascular conditions, poverty, obesity, depression, anxiety and schizophrenia (negative correlations) and HDL cholesterol levels, household income, years in education, height and head size (positive correlations)^79,80^. Little is known about the precise effects of the associated SNPs (or those they are in linkage with), although they are preferentially expressed in the central nervous system^69,70^. Two studies^73,74^ established a connection between the FNBP1L gene and general intelligence. The plexin gene family, implicated in axon guidance during neural growth, was implicated in extremely high intelligence^81^ but this effect was not replicated in the general population. The importance of neurogenesis-promoting genetic variants for general intelligence has been confirmed by a recent highly powered study^68^, which is well in line with the observed effect of intelligence in longitudinal cerebral development^82^, the stabilization of intelligence by the end of adolescent development^3,67^ and the strong genetic correlation between childhood and adult general intelligence^83,84^.

Unfortunately, if general intelligence if primarily determined by a large number of highly pleiotropic genetic variants with small individual effects, then the prospects of finding well-delineated physiological mechanisms underlying intelligence are poor in studies with sample sizes typical for behavioral neuroscience (N < 1000). However, measures which themselves reflect the functioning of complex neural systems might be exceptions as they capture the end product of complicated genetic pathways involving complementary and possible substitutable mechanisms. The sleep EEG is a good candidate for such a measure, given its high individual stability^24,27,85^, strong genetic determination^27,32,86^ and the fact that it reflects the events in large, functionally connected neural assemblies free from contamination by wakeful mentation and movement artifacts.

Our results confirm that activity in the sleep spindle-related sigma frequency range is related to intelligence. Sleep spindles are among the most genetically determined parts of the sleep EEG spectrum^86^, therefore, this association is unsurprising. However, reductions in sleep spindles are observed in a very wide variety of neurological and psychiatric conditions as well, including schizophrenia^87,88^ and Alzheimer’s disease^89^, indicating that sleep spindles resemble a non-specific marker of the integrity of the central nervous system. Future studies with available sleep EEG, intelligence and genetic data may investigate whether common variants associated with years in education^70^ or cognitive ability^69^ are also associated with sleep spindles, which would indicate that one of the effects of these variants is promoting a more efficient form of thalamocortical communication and thus propose a genetically mediated physiological mechanism of general intelligence. Alternatively, if sleep spindles are negatively associated with rare genetic variants^76–78^ then a more likely explanation is that mutational load is similar to neurological and psychiatric disease as a non-specific reducer of sleep spindle activity, and the association between sleep spindles and intelligence is also due to non-specific genetic effects. Little is known, however, about the generating mechanisms of the other oscillations we found to be associated with intelligence, therefore, their further study is recommended in order to elucidate their potential contributing mechanism to cognitive ability

## Conclusions

Overall, our results suggest that 1.) a wide range of spectral features in the sleep EEG are associated with intelligence, suggesting that the relationship between sleep EEG and IQ go well beyond sleep spindles and 2.) just like sleep spindles, other spectral characteristics of the sleep EEG are also associated with IQ in a sexually dimorphic manner, suggesting that sleep oscillations in general are more related to cognitive performance in women than men. Arguably, the vast majority of scalp EEG studies in healthy human subjects have focused on either slow waves or sleep spindles. Our results demonstrate that the potential functional relevance of other oscillations, most notably REM beta oscillations^50^, which are also trait-like and thus possible markers of individual differences, might have been underestimated. Since much research is still needed in order to understand why less-researched NREM or REM oscillations reflect individual differences, it would be important to conduct studies aimed to either replicate these findings or to elucidate the precise neurophysiology behind NREM alpha or REM beta oscillations. Similarly, future analyses are suggested for finding the potential correlates of intelligence in the sleep EEG of males. While power spectral density is not correlated with intelligence in males, other measures of sleep EEG may be. In line with wake EEG results^22,90^, EEG phase locking, coherence and regional asymmetries are suggested as candidate measures. However, our results suggest that intelligence – even if measured by the same tests – has different neural substrates in males and females.

## Electronic supplementary material

Supplementary Information
