# Supplementary material for: The sleep EEG spectrum is a sexually dimorphic marker of general intelligence
Source: Sci Rep. 2017 Dec 22;7:18070. doi: 10.1038/s41598-017-18124-0 (PMC5741768; doi:10.1038/s41598-017-18124-0)
Supplement: Supplementary file 1 — Supplementary Information [file 41598_2017_18124_MOESM1_ESM.pdf]

The sleep EEG spectrum is a sexually dimorphic marker of general intelligence

Péter P. Ujma[1], Boris N. Konrad[2], Ferenc Gombos[3], Péter Simor[4,5], Adrián Pótári[5], Lisa Genzel[2,6], Marcel Pawlowski[7], Axel Steiger[7], Róbert Bódizs[1,3]*, Martin Dresler[2]*

[1] Institute of Behavioural Sciences, Semmelweis University, H-1089 Budapest, Hungary

[2] Donders Institute for Brain, Cognition and Behaviour, Radboud University Medical Centre, 6525 EN Nijmegen, The Netherlands

[3] Department of General Psychology, Pázmány Péter Catholic University, H-1088 Budapest, Hungary

[4] Nyírő Gyula Hospital, National Institute of Psychiatry and Addictions, H-1135 Budapest, Hungary

[5] Department of Cognitive Sciences, Budapest University of Technology and Economics, H-1111 Budapest, Hungary

[6] Centre for Cognitive and Neural Systems, University of Edinburgh, EH8 9JZ Edinburg, United Kingdom

[7] Max Planck Institute of Psychiatry, 80804 Munich, Germany

* equal contribution

	N	EEG recording sites (10-20 system)	Polygraphic channels	Electrodes used	Effective sampling rate/ sampling rate (Hz)	Precision	Hardware prefiltering (Hz)	Recording apparatus	Recording software
**Budapest – I.**	24	Fp1, Fp2, F3, F4, Fz, F7, F8, C3, C4, Cz, P3, P4, T3, T4, T5, T6, O1, O2	left and right EOG, bipolar submental EMG, ECG, thoracic and abdominal respiration	Au coated Ag/AgCl fixed with EC2 Grass electrode cream	249/249	12 bit (?)	0.5-70	Flat Style SLEEP La Mont Headbox, HBX32-SLP preamplifier (La Mont Medical Inc. USA)	DataLab (Medcare, Iceland)
**Budapest – II.**	16	Fp1, Fp2, F3, F4, Fz, F7, F8, C3, C4, Cz, P3, P4, Pz, T3, T4, T5, T6, O1, O2	bipolar EOG, bipolar submental EMG, ECG	Au coated Ag/AgCl fixed with EC2 Grass electrode cream	4096/1024	12 bit	0.33-1500 (<450 Hz antialiasing digital filtering before under sampling)	Brain-Quick BQ 132S (Micromed, Italy)	System 98 (Micromed, Italy)
**Munich – I.**	91	Fp1, Fp2, AF1, AF2, F3, F4, Fz, F7, F8, C3, C4, Cz, P3, P4, Pz, T3, T4, T5, T6, O1, O2	bipolar EOG, bipolar submental EMG, ECG	Ag/Ag-Cl, with EC2 Grass Electrode Cream for EEG and Nihon Kohden ELEFIX for EMG	250/250	8 bit	0.53-70	Comlab 32 DigitalSleep Lab	Brainlab V 3.3
**Munich – II.**	20	Fp1, Fp2, F3, F4, C3, C4, P3, P4, O1, O2	bipolar EOG, ECG	Ag/Ag-Cl, with EC2 Grass Electrode Cream for EEG and Nihon Kohden ELEFIX for EMG	250/250	8 bit	0.53-70	Comlab 32 DigitalSleep Lab	Brainlab V 3.3

Supplementary Table 1. Details of the recording procedures in different subsamples.

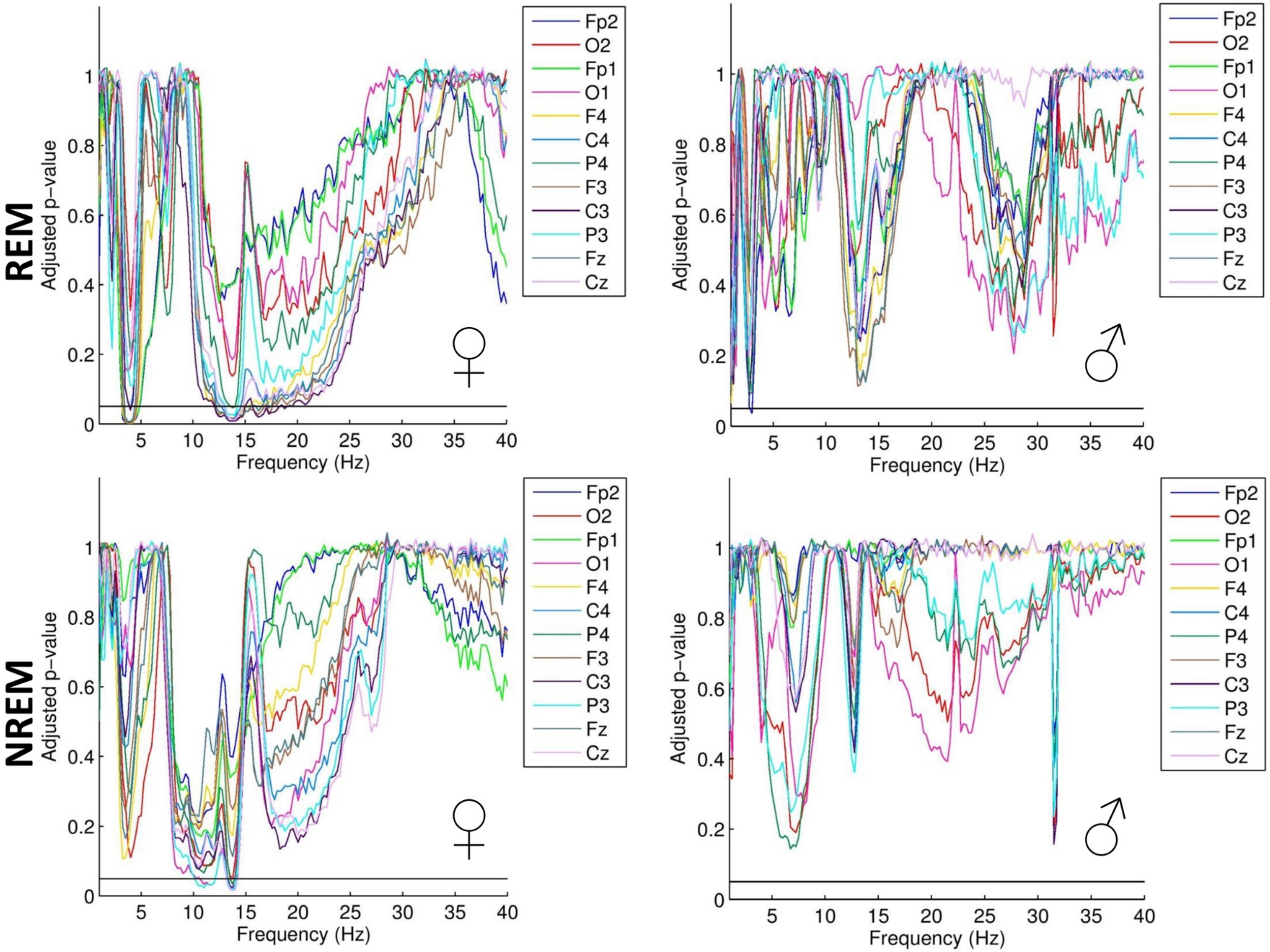

Supplementary Figure 1. Results of the permutation bootstrapping method of multiple comparisons correction. Adjusted p-values between 1 and 40 Hz are shown for all midline electrodes, separately for REM sleep (upper panels), NREM sleep (lower panels) as well as for females (left panels) and males (right panels). A vertical line parallel to axis x marks p adjusted =0.05 in order to highlight associations which remained significant after multiple comparisons correction.
